# Suppressive effects of S100A8 and S100A9 on neutrophil apoptosis by cytokine release of human bronchial epithelial cells in asthma

**DOI:** 10.7150/ijms.37833

**Published:** 2020-02-04

**Authors:** Da Hye Kim, Ayoung Gu, Ji-Sook Lee, Eun Ju Yang, Ayesha Kashif, Min Hwa Hong, Geunyeong Kim, Beom Seok Park, Soo Jin Lee, In Sik Kim

**Affiliations:** 1 Department of Senior Healthcare, BK21 Plus Program, Graduate School, Eulji University, Daejeon 34824; 2 Department of Clinical Laboratory Science, Wonkwang Health Science University, Iksan, 54538; 3 Department of Clinical Laboratory Science, Daegu Haany University, Gyeongsan, 38610; 4 Department of Biomedical Laboratory Science, College of Health Science, Eulji University, Seongnam 13135; 5 Department of Pediatrics, School of Medicine, Eulji University, Daejeon, 301-746; 6 Department of Biomedical Laboratory Science, School of Medicine, Eulji University, Daejeon 34824, Republic of Korea

**Keywords:** S100A8, S00A9, Asthma, Neutrophil apoptosis, Cytokine

## Abstract

S100A8 and S100A9 are important proteins in the pathogenesis of allergy. Asthma is an allergic lung disease, characterized by bronchial inflammation due to leukocytes, bronchoconstriction, and allergen-specific IgE. In this study, we examined the role of S100A8 and S100A9 in the interaction of cytokine release from bronchial epithelial cells, with constitutive apoptosis of neutrophils. S100A8 and S100A9 induce increased secretion of neutrophil survival cytokines such as MCP-1, IL-6 and IL-8. This secretion is suppressed by TLR4 inhibitor), LY294002, AKT inhibitor, PD98059, SB202190, SP600125, and BAY-11-7085. S100A8 and S100A9 also induce the phosphorylation of AKT, ERK, p38 MAPK and JNK, and activation of NF-κB, which were blocked after exposure to TLR4i, LY294002, AKTi, PD98059, SB202190 or SP600125. Furthermore, supernatants collected from bronchial epithelial cells after S100A8 and S100A9 stimulation suppressed the apoptosis of normal and asthmatic neutrophils. These inhibitory mechanisms are involved in suppression of caspase 9 and caspase 3 activation, and BAX expression. The degradation of MCL-1 and BCL-2 was also blocked by S100A8 and S100A9 stimulation. Essentially, neutrophil apoptosis was blocked by co-culture of normal and asthmatic neutrophils with BEAS-2B cells in the presence of S100A8 and S100A9. These findings will enable elucidation of asthma pathogenesis.

## Introduction

S100A8 and S100A9 are crucial members of the calcium-binding S100 protein family, mainly expressed in neutrophils and monocytes 1, 2. Extracellular functions of S100A8 and S100A9 include modulating pro-inflammatory responses such as cytokine secretion, leucocyte recruitment, apoptosis and tissue damage 3. S100A8 and S100A9 trigger the expression of IL-6 and MCP-1, and the release of CXCL10, from human umbilical vein endothelial cells or macrophages 1, 4. Cytokine release induced by S100A8 and S100A9 plays a specifically important role in inflammatory responses. S100A8 and S100A9 are considered as damage-associated molecular patterns (DAMPs) recognized by Toll-like receptor (TLR4) or receptor for advanced glycation end products (RAGE). Therefore, S100A8 and S100A9 are essential causative stimulators in inflammatory diseases, and may be used as biomarkers and therapeutic targets 5-7.

Lung infiltration by neutrophils is a prominent phenomenon in neutrophilic asthma. Inhibition of neutrophil apoptosis prolongs the neutrophil survival, which aggravates clinical features of asthma by maintaining inflammatory responses 8, 9. Neutrophil apoptosis is regulated by a variety of cytokines such as IL-6, IL-8, GM-CSF, G-CSF, and MCP-1 10-12. Airway epithelial cells play important roles in pathogenesis of asthma as structural cells. The relationship of airway epithelial cells and asthma differs, depending on the allergen, virus infection and effective mechanisms 13-15. Nevertheless, understanding the interaction of immune cells and non-immune cells is essential in unveiling the pathogenic mechanism of asthma and developing a therapeutic strategy 16.

Our previous paper demonstrates that S100A8 and S100A9 in BALF and serum of asthma subjects are significantly related to increased serum IgE, and they directly inhibit the neutrophil apoptosis via TLR4, Lyn, PI3K, AKT, ERK, and NF-κB 17. In this study, we investigate whether S100A8 and S100A9 induce the cytokine secretion of airway epithelial cells, which affects neutrophil apoptosis of normal and asthmatic subjects.

## Materials and methods

### Materials

DMEM/F12, RPMI 1640, penicillin and streptomycin solution, trypsin/EDTA and TRIzol reagent were obtained from Life Technologies Inc. (Gaithersburg, MD, USA). Fetal bovine serum (FBS) was acquired from RMBIO (Missoula, MT, USA). *Dermatophagoides pteronissinus* (DP) extract was purchased from Cosmo Bio (Tokyo, Japan). TLR4 inhibitor (TLR4i), CLI-095, was purchased from Invivogen (San Diego, CA, USA). Inhibitors for PI3K (LY294002), AKT (AKTi), ERK (PD98059), p38 MAPK (SB202190), JNK (SP600125) and NF-κB (BAY-11-7085) were obtained from Calbiochem (San Diego, CA, USA). Antibodies against-phospho-AKT, AKT, ERK2, TLR4, and BCL2 were obtained from Santa Cruz Biotechnology (Santa Cruz, CA, USA). Anti-phospho-p38 MAPK, anti-phospho-ERK1/2, anti-phospho-JNK, anti-p38 MAPK, anti-JNK, anti-cleaved caspase 9, anti-cleaved caspase 3, BAX, and MCL-1 were procured from Cell Signaling Technology (Beverly, MA, USA).

### Cell culture

The human bronchial epithelial cell line, BEAS-2B, transformed with an adenovirus 12-SV40 virus hybrid (CRL-9609; ATCC, Manassas, VA, USA), was cultured in DMEM/F12 medium supplemented with 10% heat-inactivated fetal bovine serum (FBS), penicillin (100 U/mL), and streptomycin (100 μg/mL).

### Production of recombinant proteins

Briefly, total RNA of DP was extracted using the TRIzol reagent and purified by an RNeasy Mini Kit (Qiagen, Hilden, Germany), followed by synthesis of cDNA. Der p 38 cDNA was amplified by PCR using forward primer (5'- ACT ACG GAT CCG ATG AAT GGT GCC GCT ATT-3') and reverse primer (5'- ACT ACG CGG CCG CTC ACC AAC ATC GTG CAA CAT TAG C-3'). The PCR product was cut with BamHI and NotII (New England Biolabs, Ipswich, MA, USA) and cloned into the pETDuet-1 vector (Merck Millipore, Darmstadt, Germany). His-Tagged Der p 38 recombinant protein was expressed in *E. coli* BL21(DE3) cells, followed by separation using a nickel column (Merck Millipore, Darmstadt, Germany). Finally, the protein was purified on a Superdex 200 column attached to an ÄKTA FPLC system (GE Healthcare). Recombinant S100A8 and S100A9 proteins were produced as previously reported 17. Briefly, cDNAs were synthesized by reverse transcription of total RNA of human neutrophils. The amplified cDNAs of S100A8 and S100A9 were cloned into the pET28 vector (Merck Millipore). Recombinant His-Tag S100A9 was induced and then purified using a nickel column. The purified proteins were identified by western blotting using anti-S100A8, anti S100A9, and Der p 38 antibodies.

### Enzyme-linked immunosorbent assay (ELISA)

Concentrations of IL-6, IL-8, MCP-1 and GM-CSF in the cell supernatant were evaluated with a sandwich ELISA. Human GM-CSF Quantikine (R&D systems, Minneapolis, MN, USA) and OptEIA^TM^ Set ELISA kit (BD Biosciences, San Diego, CA, USA) were used for detection of GM-CSF and human IL-6, IL-8, MCP-1, respectively, according to the manufacturer's manual.

### Western blotting

Cells were lysed in cytosolic lysis buffer (TransLab, Daejeon, Korea). After centrifugation, the supernatant was collected and protein concentration of the lysate was measured by a protein assay kit (Thermo scientific, Waltham, MA, USA). Protein samples were loaded on 10% SDS-PAGE gel, separated by electrophoresis and transferred to nitrocellulose filters. The membranes were then probed with primary antibodies, after which the blots were incubated with appropriate secondary antibodies, including goat anti-mouse or rabbit antibodies. The blots were developed with the enhanced chemiluminescence detection system (Amersham Pharmacia Biotech.), and detected by Chemi-Doc TM touch imaging system (Bio-Rad, Richmond, CA, USA).

### NF-κB p65 transcription factor assay

DNA-binding activity of NF-κB was evaluated using the EZ-Detect^TM^ transcription factor kits for NF-κB p65 (PIERCE, Rockford, IL, USA), according to the manufacturer's instructions. Both wild type and mutant NF-κB oligonucleotides were used as negative controls. Chemiluminescent detection was performed using a luminometer.

### Detection of TLR4 and apoptosis

BEAS-2B cells were treated with S100A8 and S100A9 in a time-dependent manner. The cells were collected and washed with PBS three times. To prevent non-specific binding of antibody, the cells were incubated with 0.5% BSA blocking solution. After incubation with antibody against TLR4, cells were washed and incubated with FITC-labeled secondary antibody. Baseline fluorescence was acquired by adding normal mouse IgG instead of primary antibody. The annexin V-fluorescein isothiocyanate (FITC) apoptosis detection kit (BD Biosciences) was used to determine apoptosis of neutrophils and eosinophils. Briefly, isolated neutrophils and eosinophils were treated with the S100A8- or S100A9-stimulated supernatant, followed by subsequent incubation with FITC-labeled annexin V and propidium iodide (PI) for 15 min at room temperature. The resultant fluorescent intensity of the cells was analyzed by the FACSort cytofluorimeter (BD Biosciences). Annexin V+/PI- and annexin V+/PI+ cells are considered as apoptotic cells.

### Isolation of neutrophils and eosinophils

Human neutrophils and eosinophils were isolated from peripheral blood of normal and asthmatic subjects using Ficoll-Hypaque solution and a CD16 microbeads magnetic cell sorting kit (Miltenyi Biotec, Bergisch Gladbach, Germany). After removal of erythrocytes by hypotonic lysis buffer, the neutrophils were washed with PBS three times and cultured in RPMI 1640 containing 10% fetal bovine serum (FBS). This study was approved by the Institutional Review Board of Eulji University for normal volunteers, and by the Institutional Review Board of Eulji University Hospital for asthmatic patients. Written informed consent was received from study volunteers before blood collection.

### Statistical analysis

Data are presented as the means ± SD. A paired t-test and one-way ANOVA was used for comparing two-groups and more than two groups, respectively, using the SPSS statistical software (Chicago, IL, USA). *p* <0.05 is considered significant.

## Results

### S100A8 and S100A9 stimulate the secretion of MCP-1, IL-6, and IL-8 through TLR4, PI3K, AKT, MAPKs, and NF-κB in BEAS-2B cells

**Figure 1:**
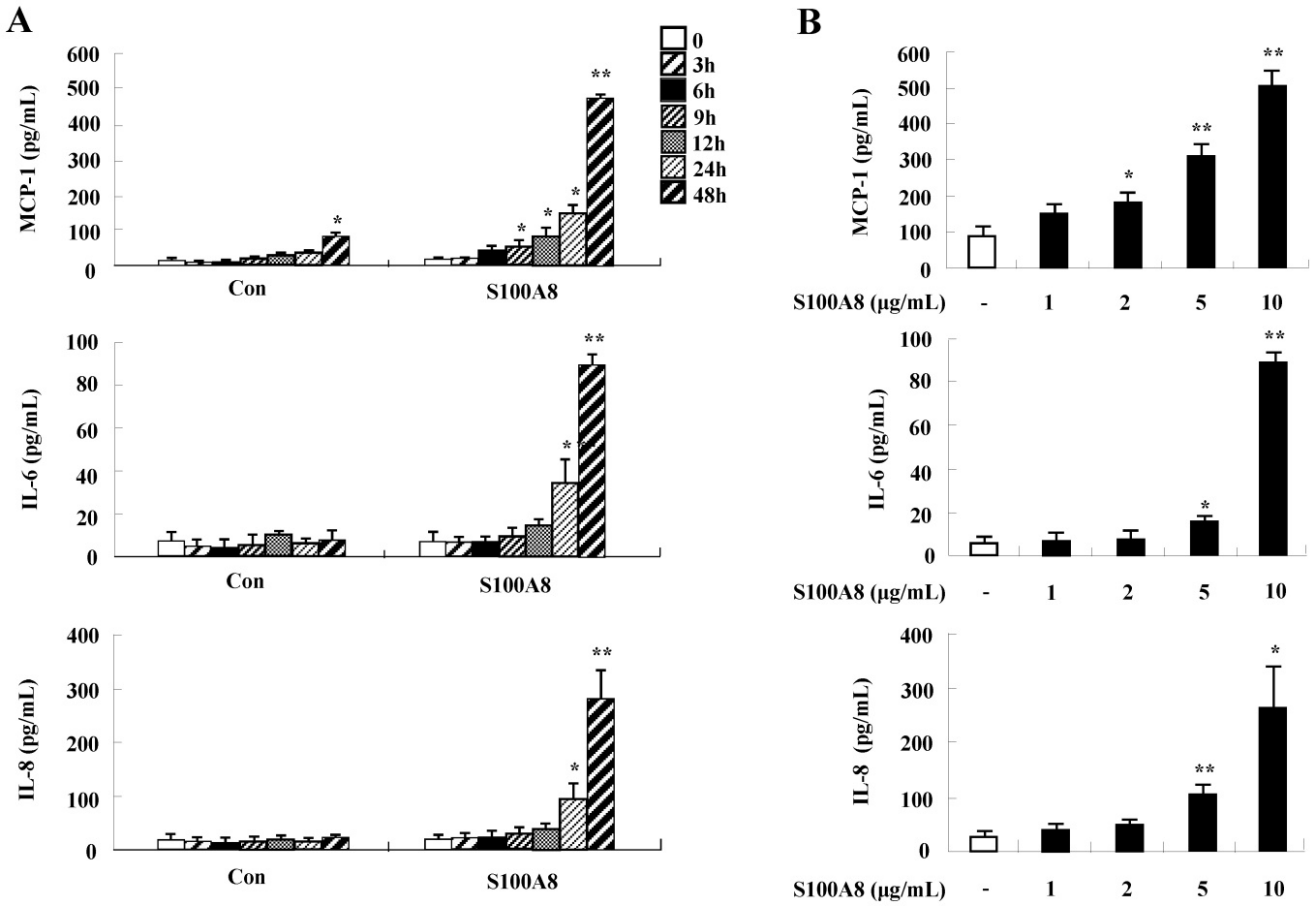

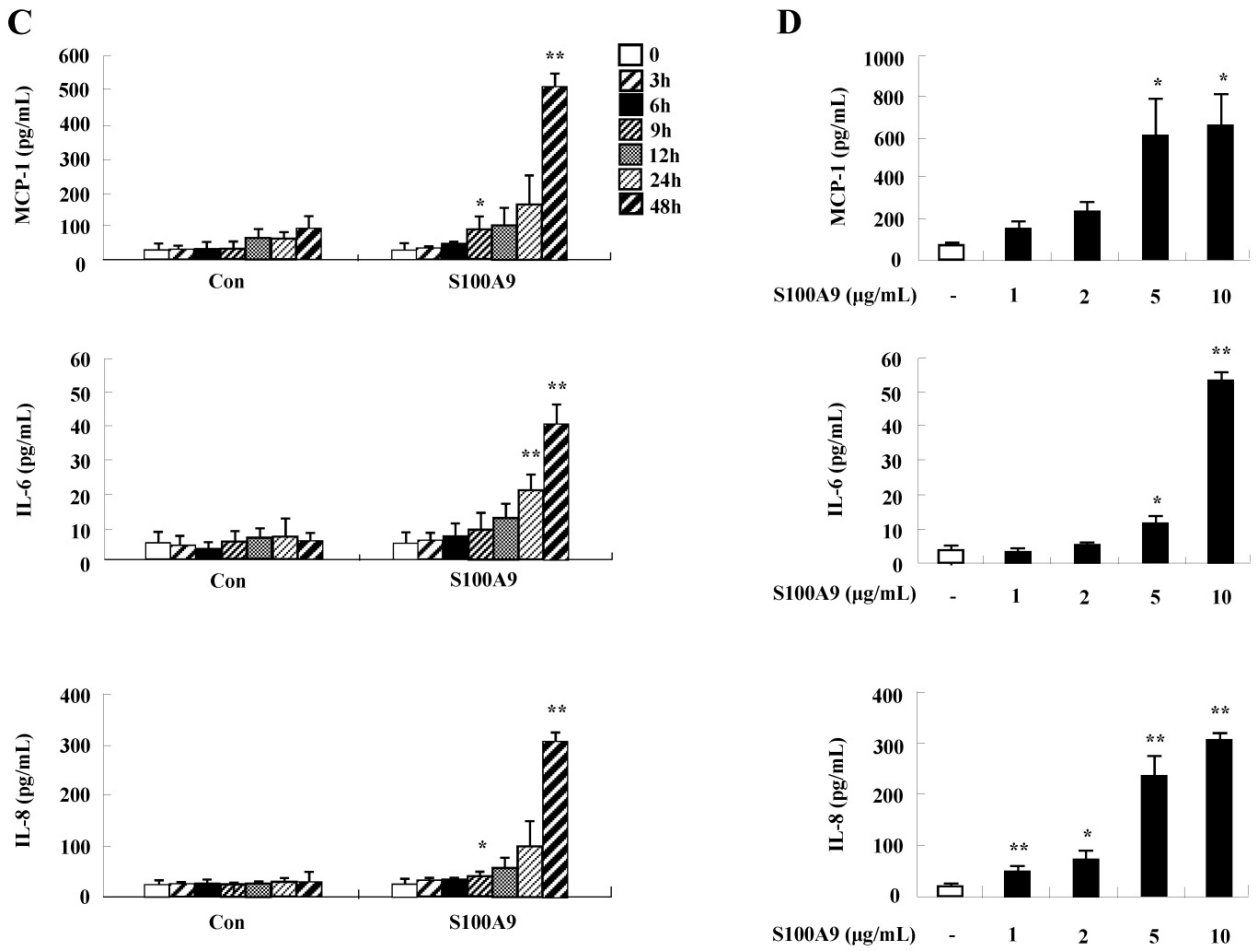
** S100A8 and S100A9 increase the production of MCP-1, IL-6, and IL-8 in BEAS-2B cells.** (A-D) BEAS-2B cells were incubated with or without 10 μg/mL S100A8 (A) and S100A9 (C) for the indicated time, or at the indicated concentration of S100A8 (B) and S100A9 (D) for 48 h. ELISA was used to determine the concentrations of MCP-1, IL-6, and IL-8 in the collected supernatant. Data are expressed as the means ± SD and are presented relative to the control. *p < 0.05 and **p < 0.01 indicate a significant difference between the control and S100A8- or S100A9-treated groups.

**Figure 2:**
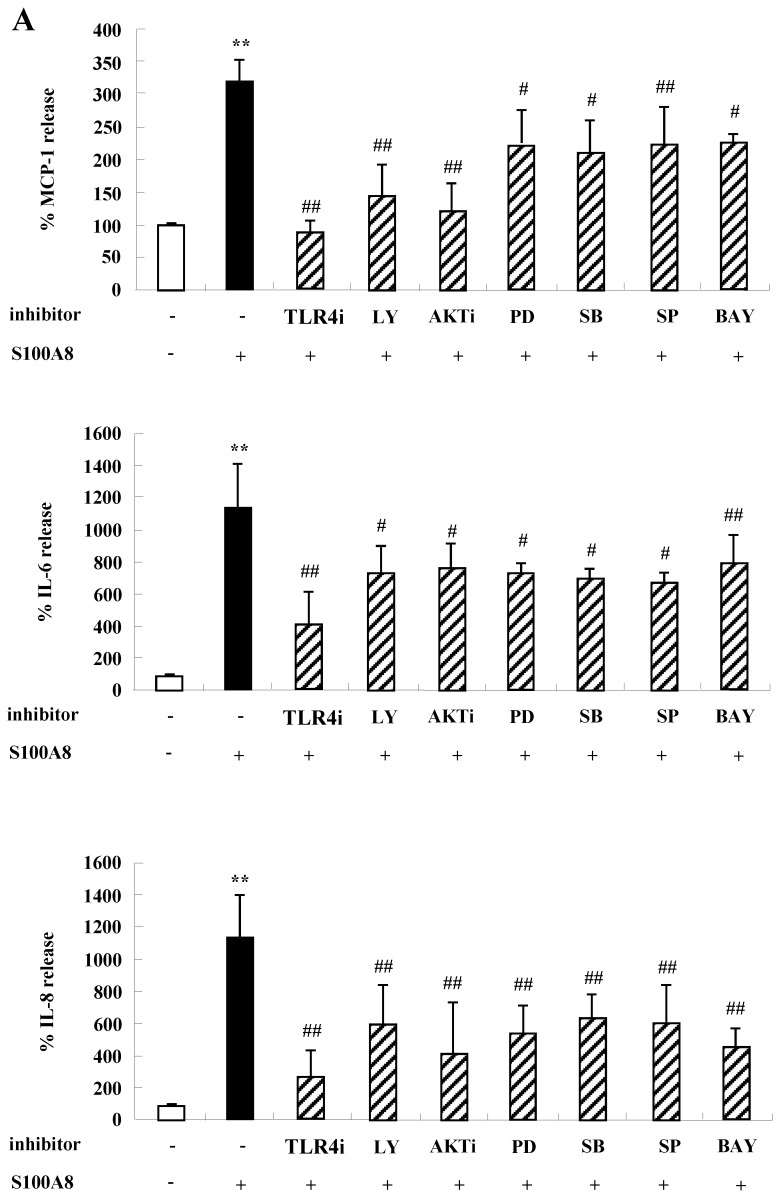

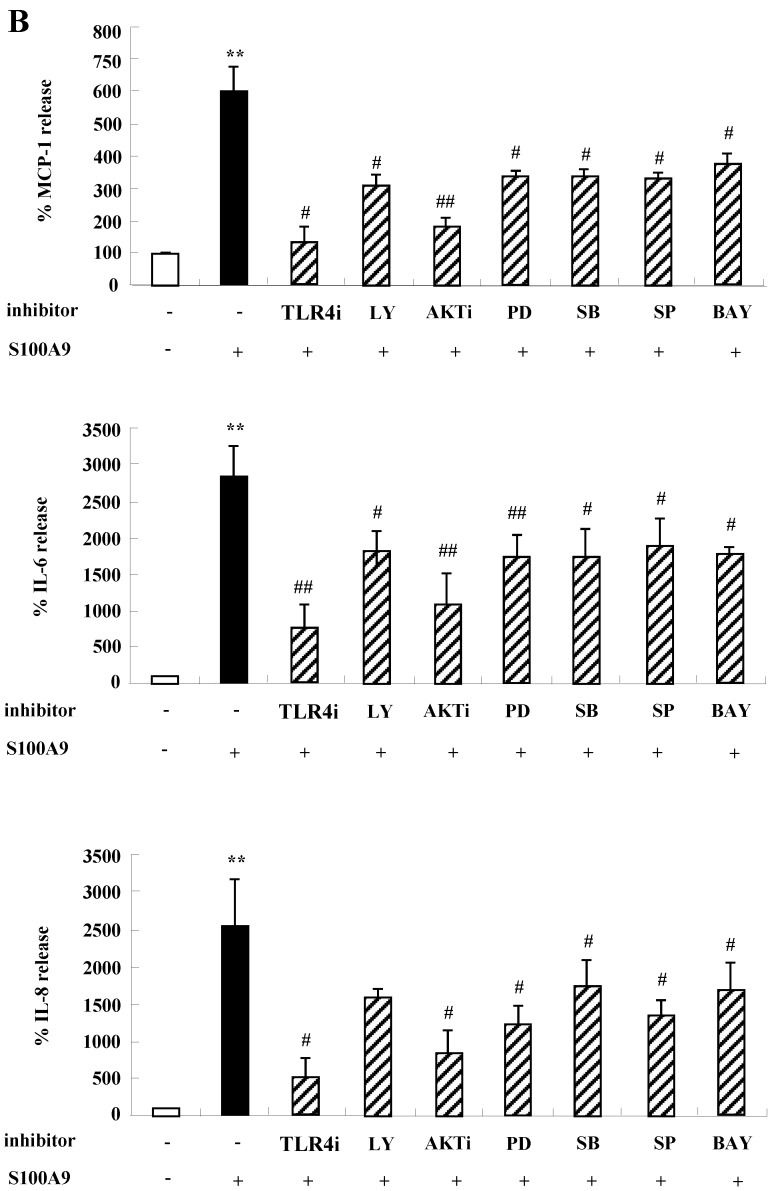
** S100A8 and S100A9 induce the secretion of MCP-1, IL-6 and IL-8 through TLR4, PI3K, AKT, MAPKs and NF-κB in BEAS-2B cells.** (A-B) BEAS-2B cells were pre-treated for 1 h with or without 5 μM TLR4i, 5 μM LY294002 (LY), 10 μM AKTi (AKT), 10 μM PD98059 (PD), 10 μM SB202190 (SB), 10 μM SP600125 (SP) and 2 μM BAY-11-7085 (BAY), after which the cells were incubated for 48 h in the absence or presence of 10 μg/mL S100A8 (A) and S100A9 (B). The supernatant (n=4) was collected and analyzed by ELISA. Data are expressed as the means ± SD and are presented relative to the control set at 100%. *p < 0.05 and **p < 0.01 indicate a significant difference between the control and S100A8- or S100A9-treated groups, and^ ##^*p* < 0.01 and ^#^*p* < 0.05 represent a significant difference between the S100A8- or S100A9-treated groups and the inhibitor-treated groups.

**Figure 3:**
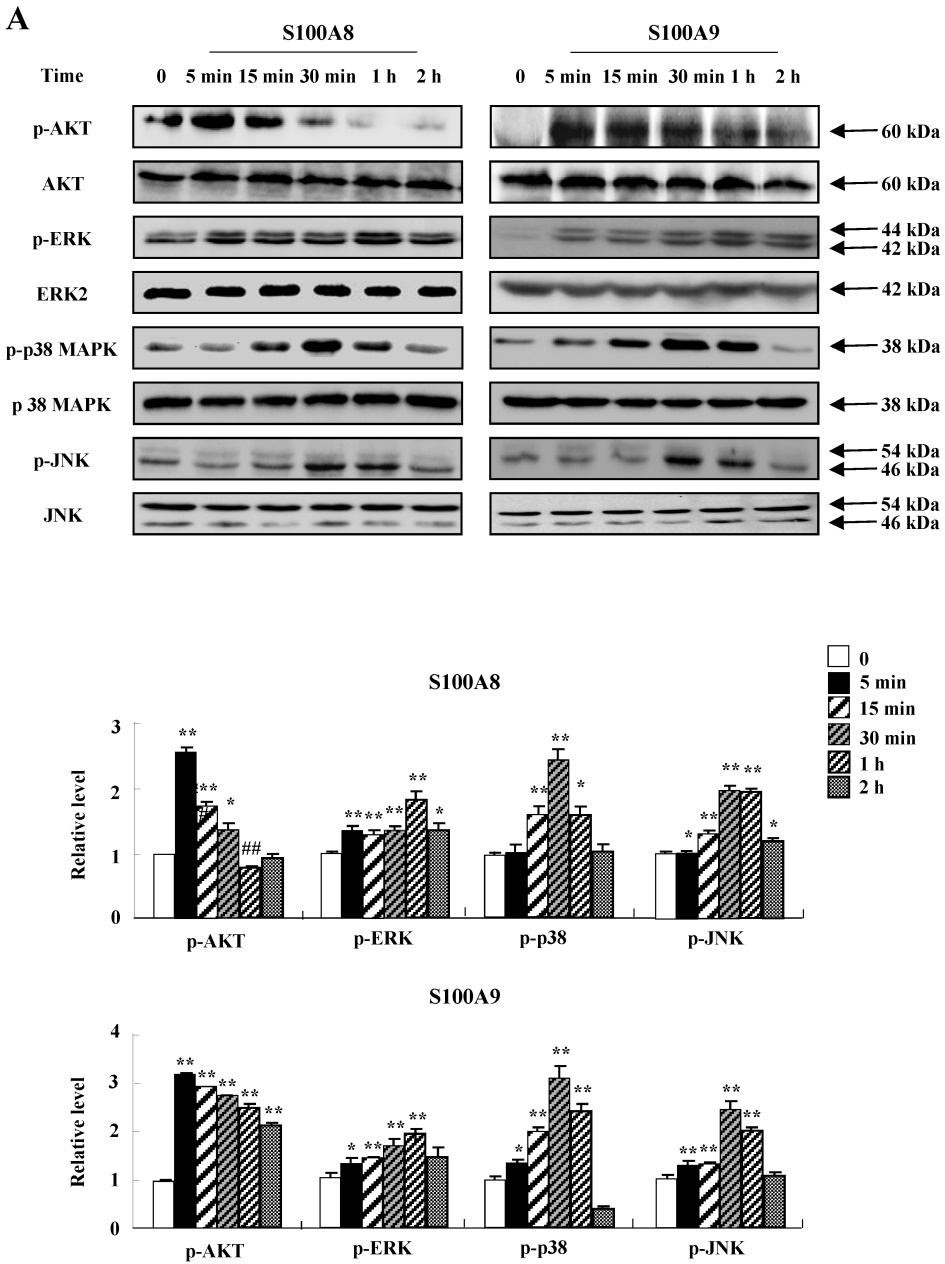

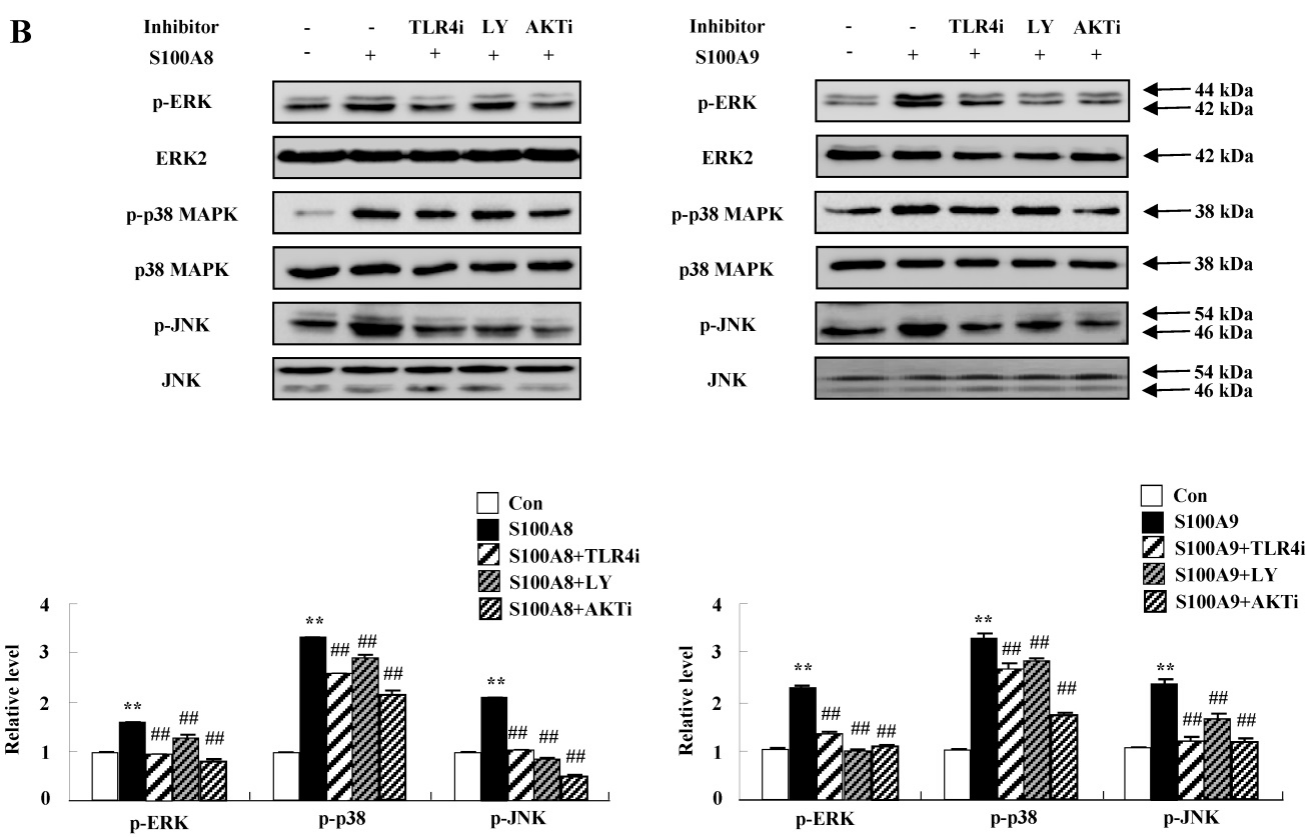

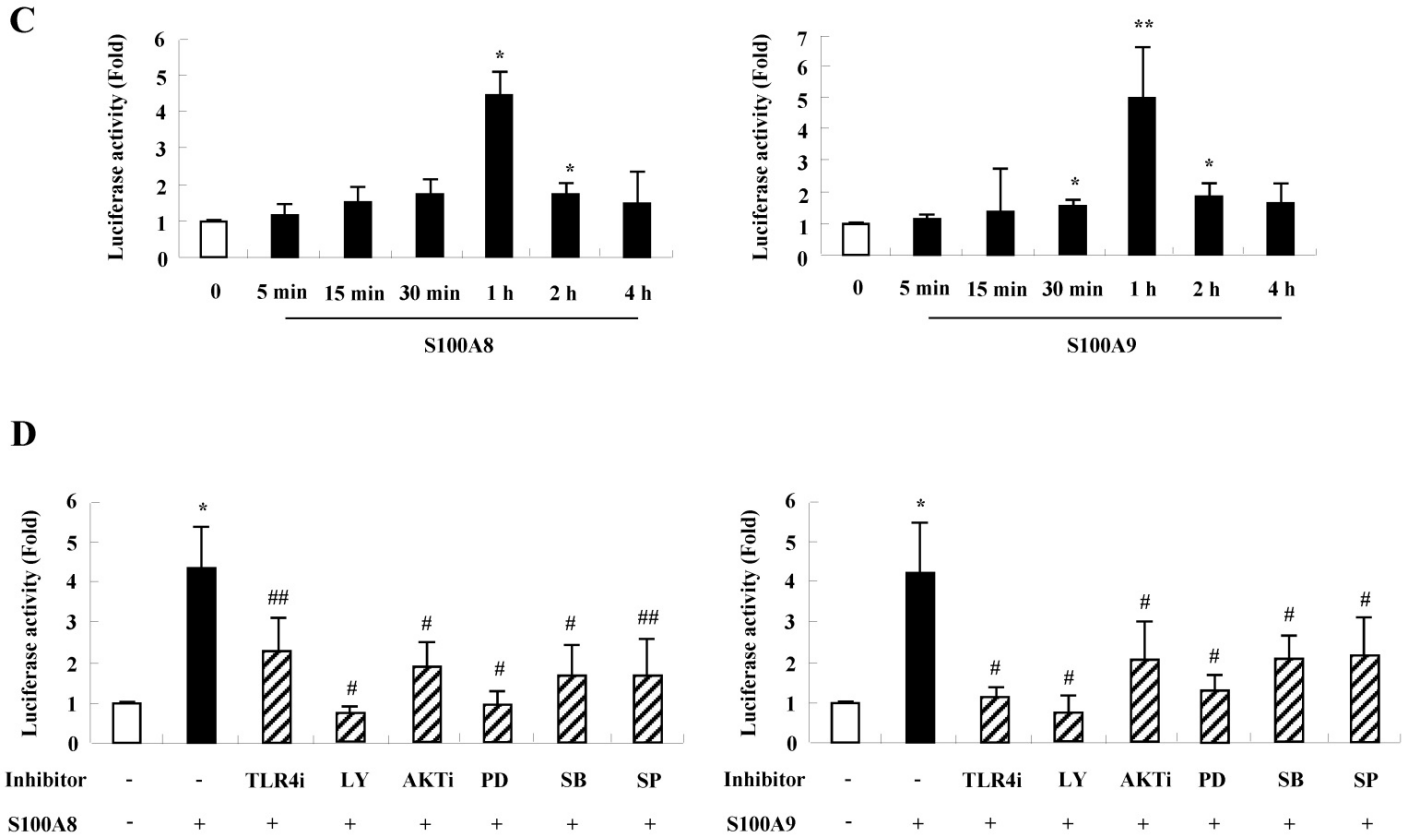
** S100A8 and S100A9 require activation of PI3K/AKT/ MAPKs/NF-κB pathway for cytokine expression.** (A) BEAS-2B cells were incubated with 10 μg/mL S100A8 (left panel) and S100A9 (right panel) for the indicated time. Phosphorylation of AKT, ERK, p38 MAPK and JNK in the lysates was detected by Western blotting. (B) BEAS-2B cells were pre-treated for 1 h with or without 5 μM TLR4i, 5 μM LY294002 (LY) and 10 μM AKTi, after which the cells were incubated with 10 μg/mL S100A8 (left panel) and S100A9 (right panel) for 30 min. After harvested cells were lysed, the phospho-ERK (p-ERK), phospho-p38 MAPK (p-p38 MAPK) and phospho-JNK (p-JNK) in the lysates were detected by Western blotting. Densitometric data are presented relative to the negative control set at l (lower panel). (C, D) BEAS-2B cells were incubated with S100A8 and S100A9 (10 μg/mL) for the indicated time (C), or were pre-treated for 1 h with or without 5 μM TLR4i, 5 μM Ly294002 (LY) and 10 μM AKTi, 10 μM PD98059 (PD), 10 μM p38 MAPK (SB) and 10 μM JNK600125 (SP), after which the cells were incubated with S100A8 and S100A9 (10 μg/mL) for 1 h (D). After collecting nuclear extract from harvested cells, NF-κB in the lysates (n=3) was detected by luciferase assay. Data are presented relative to the control set at 1, as the means ± SD. *p < 0.05 and **p < 0.01 indicate a significant difference between the control and S100A8 and S100A9-treated groups, and^ ##^*p* < 0.01 and ^#^*p* < 0.05 represent a significant difference between the S100A8- or S100A9-treated and inhibitor-treated groups.

To unveil more specific inflammatory action due to S100A8 and S100A9, we examined the secretion of cytokines in BEAS-2B cells, particularly the neutrophil survival cytokines. We observed increased levels of MCP-1, IL-6, and IL-8 after treatment with S100A8 and S100A9, in a time- and dose-dependent manner (Fig. 1). To investigate specific mechanisms of S100A8 and S100A9 on cytokine secretion, we examined alteration of cytokine production after signal inhibitor treatment. Inhibitors used in this study do not solely affect the release of MCP-1, IL-6, and IL-8 in BEAS-2B cells and (Supple. Fig. 1). TLR4i, LY294002, PD98059, SB202190, SP600125, and BAY-11-7085 strongly blocked the increased secretion of MCP-1, IL-6, and IL-8 induced by S100A8 and S100A9, despite different degrees of inhibitory effects (Fig. 2A and B). S100A8 and S100A9 induced the phosphorylation of AKT and MAPKs in a time-dependent manner (Fig. 3A). Activation of ERK, p38 MAPK, and JNK due to S100A8 and S100A9 was suppressed by TLR4i, LY294002, and AKTi (Fig. 3B). As shown in Fig. 3C and D, NF-κB activation occurs after exposure to S100A8 and S100A9, and peaks at 1 h. The movement of NF-κB to nucleus was blocked by TLR4i, LY294002, AKTi, PD98059, SB202190, and SP600125.

### TLR4 expression increases after treatment with S100A8 and S100A9 in BEAS-2B cells

**Figure 4:**
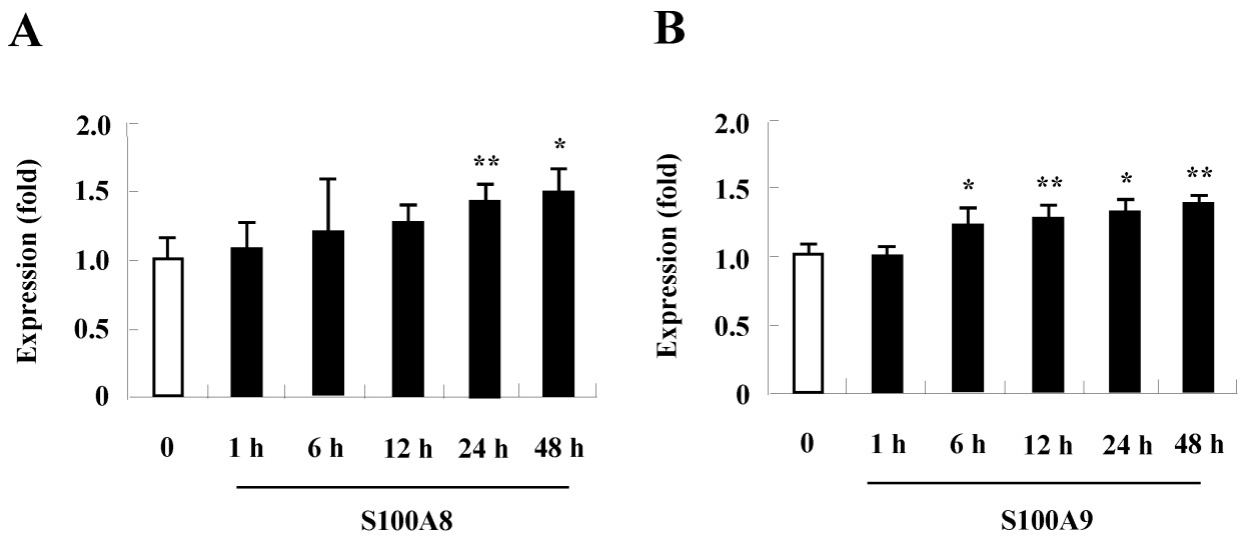
** Expression of TLR4 in BEAS-2B cells after treatment with S100A8 and S100A9.** BEAS-2B cells (n=3) were treated with 10 μg/mL S100A8 (A) and S100A9 (B) for the indicated time, and the expression of TLR4 was analyzed by flow cytometry. Data are expressed as the means ± SD, and are presented relative to the control set at 1. *p < 0.05 and **p < 0.01 indicate a significant difference.

Since change of receptor expression affects the signal transduction that occurs after exposure of its stimulator, examining whether TLR4 expression is altered by treatment with S100A8 and S100A9 treatment revealed that S100A8 and S100A9 gradually augment TLR4 surface expression in a time-dependent manner (Fig. 4). These results indicate that S100A8 and S100A9 continue to strongly induce their responses by increasing TLR4 expression.

### Cytokine release (MCP-1, IL-6, and IL-8) by S100A8 and S100A9 inhibits apoptosis of normal and asthmatic neutrophils

**Figure 5:**
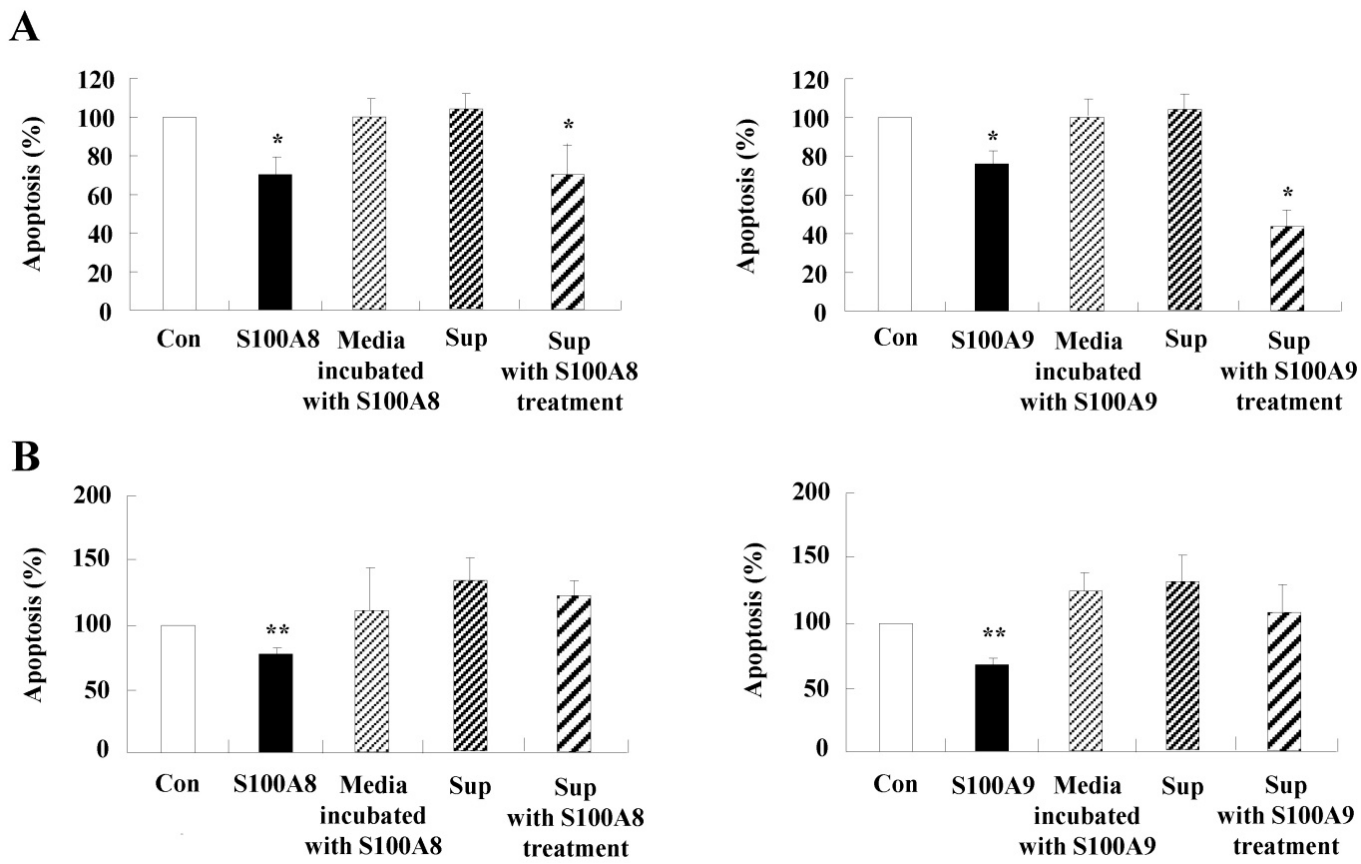

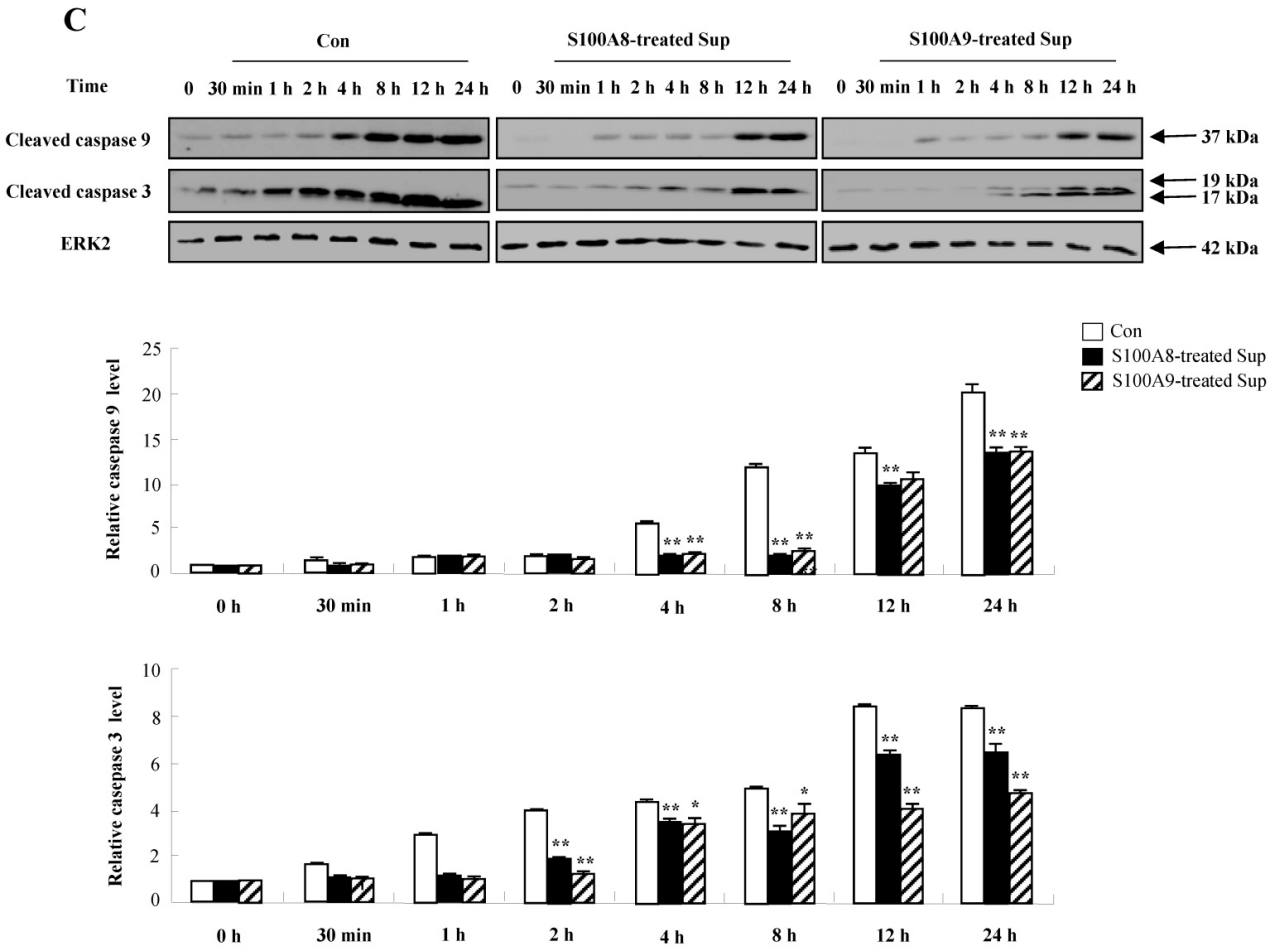

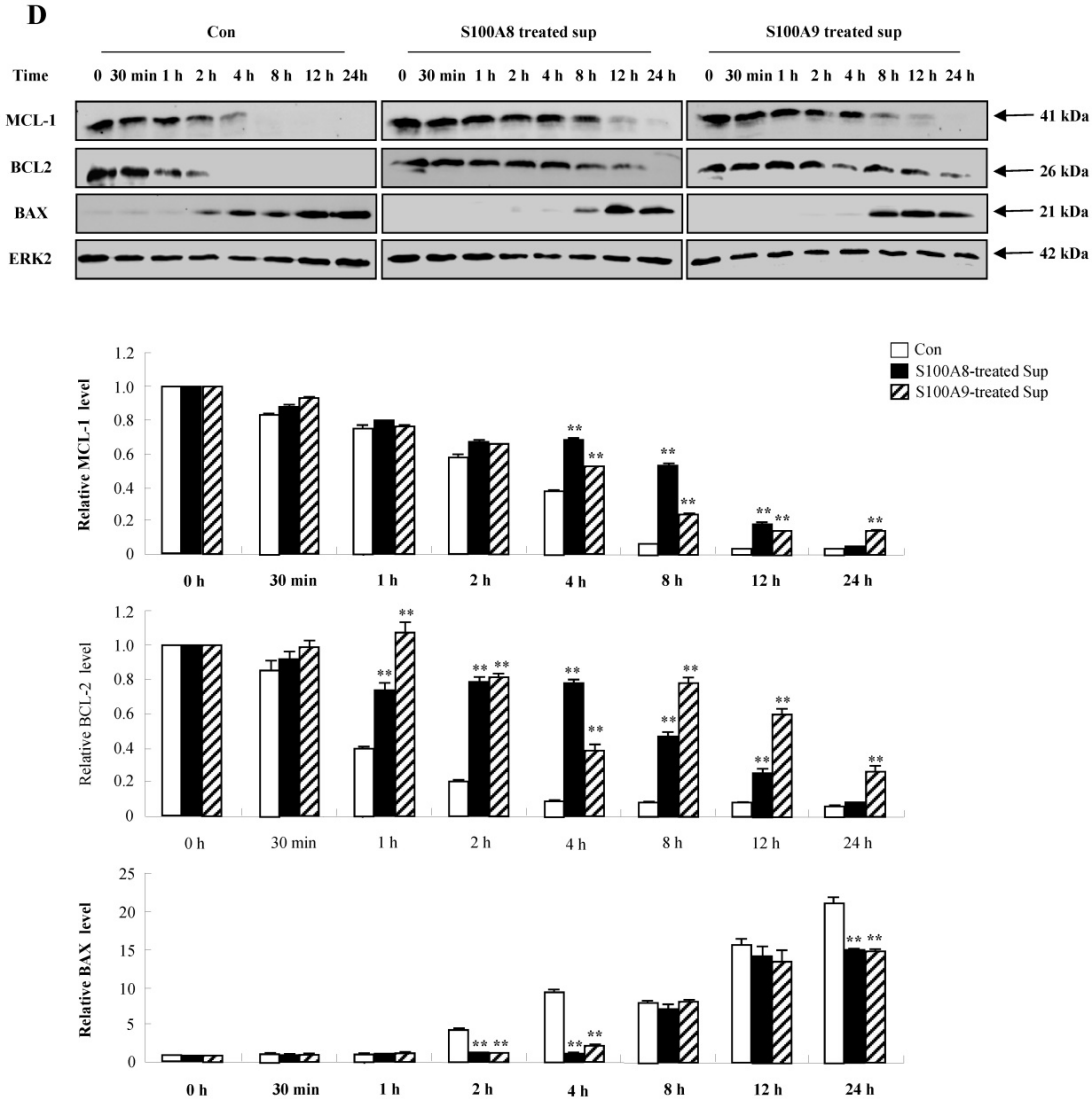

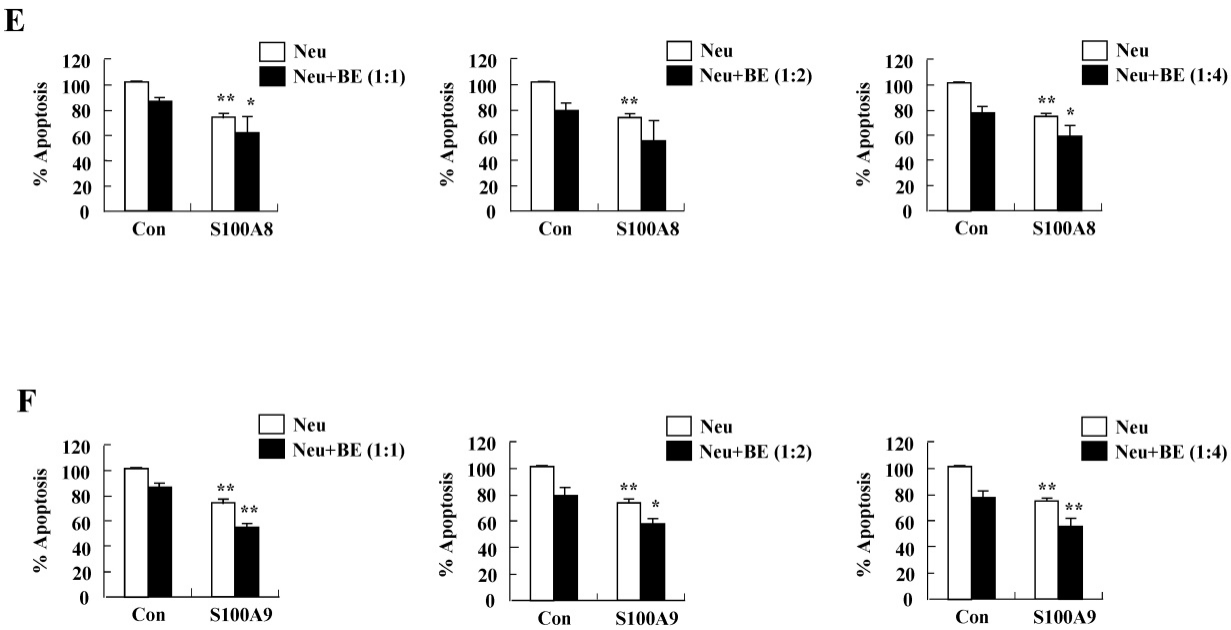
** Constitutive apoptosis of normal neutrophils is delayed by released molecules due to S100A8 and S100A9 in BEAS-2B cells.** (A, B) BEAS-2B cells were incubated with or without 10 μg/mL of S100A8 and S100A9 for 48 h. The supernatant (Sup) was collected and added to the fresh neutrophils (A) (n=3) and eosinophils (B) (n=3) isolated from peripheral blood. Apoptosis of neutrophils and eosinophils was evaluated by measuring the binding of annexin V-FITC and PI. (C, D) Neutrophils were incubated with the S100A8 and S100A9-treated supernatant (Sup) of BEAS-2B cells. Expression levels of cleaved caspase 9 and caspase 3 (C), and MCL-1, BCL-2, and BAX (D) were detected by Western blotting. Densitometric data are presented relative to the negative control, set at 1 (lower panel). (E, F) Normal neutrophils (n=4) and BEAS-2B cells (1:1, 1:2, and 1:4 ratio) were incubated for 24 h in the absence or presence of S100A8 (E) and S100A9 (F) (10 μg/mL). Neutrophil apoptosis was analyzed by measuring the binding of annexin V-FITC and PI. Data are presented relative to the control, set at 100%, as the means ± SD. *p < 0.05 and **p < 0.01 indicates a significant difference between the control and S100A8- or S100A9 treated groups.

**Figure 6:**
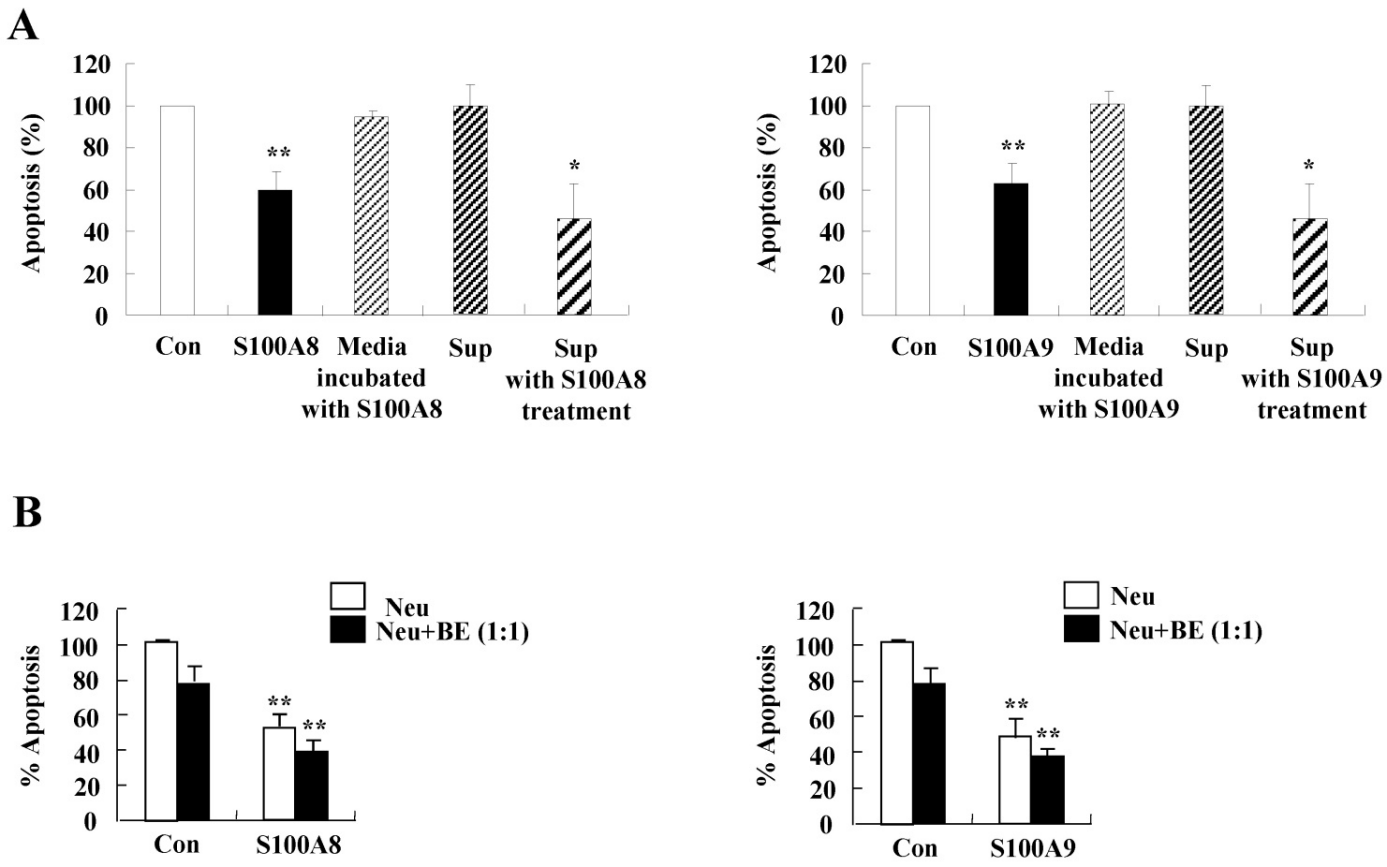
** The secretion of cytokines induced by S100A8 and S100A9 in BEAS-2B cells inhibits asthmatic neutrophil apoptosis.** (A) BEAS-2B cells were incubated with or without 10 μg/mL of S100A8 and S100A9 for 48 h. The supernatant (Sup) was collected and added to the fresh neutrophils isolated from the peripheral blood of asthmatic subjects (n=5). (B) Asthmatic neutrophils or neutrophils and BEAS-2B cells (1:1) were incubated for 24 h in the absence or presence of 10 μg/mL S100A8 (left panel) and S100A9 (right panel). Apoptosis of asthmatic neutrophils was analyzed by measuring the binding of annexin V-FITC and PI. Data are presented relative to the control set at 100%, as the means ± SD. *p < 0.05 and **p < 0.01 indicates a significant difference between the control and S100A8- or S100A9-treated groups, or between the control supernatant and supernatant-treated groups.

We investigated whether cytokines secreted by S100A8 and S200A9 alter the spontaneous neutrophil apoptosis. In normal subjects, the supernatant treated with S100A8 or S100A9 inhibits the neutrophil apoptosis (Fig. 5A), but is not effective on eosinophil apoptosis (Fig. 5B). Cytokines released by S100A8 and S100A9 suppress the activation of caspase 9 and caspase 3 (Fig. 5C). The expression of MCL-1 and BCL2 shows less decrease, and BAX expression shows less increase in BEAS-2B cells after exposure to the supernatant treated with S100A8 and S100A9 as compared to the control (Fig. 5D). Coculture of BEAS-2B cells with neutrophils reveals suppression of neutrophil apoptosis after treatment with S100A8 and S100A9, which is comparable to the inhibitory effect of S000A8 and S100A9 on neutrophil apoptosis (Fig. 5E and F). Furthermore, BEAS-2B cells cocultured with neutrophils inhibited the neutrophil apoptosis without treatment. In asthmatic subjects, the S100A8- and S100A9-stimulated supernatant also blocked neutrophil apoptosis (Fig. 6A). Neutrophils cocultured with BEAS-2B cells stimulated with S100A8 and S100A9 underwent less apoptosis compared to the control (Fig. 6B).

## Discussion

Sterile inflammation is an inflammatory response which appears in our body without microbial components, and is related to allergies, autoimmune diseases and other inflammatory diseases 18, 19. S100 proteins, also considered as DAMP, have been focused in the field of cancer as well as inflammatory diseases. In the present study, we demonstrate that cytokine release in bronchial cells is induced by S100A8 and S100A9, and this reaction prolongs the inflammatory responses by inhibition of neutrophil apoptosis in normal and asthmatic subjects. We believe that the inflammatory response due to S100A8 and S100A9 is a critical factor in asthma pathogenesis.

Both S100A8 and S100A9 are essential proteins of the S100 family proteins and play important roles in allergies, such as asthma 20. Our previous and present results reveal that neutrophil apoptosis is suppressed by not only S100A8 and S100A9 but also the secondary response, namely, cytokine release secreted by S100A8 and S100A9 17 (Fig. 1). Bronchial epithelial cells are important cytokine producers. As shown in Figs. 1, 5, and 6, they enhance the production of MCP-1, IL-6, and IL-8, resulting in the subsequent suppression of neutrophil apoptosis. Bronchial cells express various cytokines that regulate the Th1, Th2, and Th17 immune responses 21, 22. A recent study reported that S100A9 triggers the development of neutrophilic inflammation by elevation of IL-1β, IL-17 and IFN-γ, and induces allergen-induced Th2 type inflammation by production of thymic stromal lymphopoietin (TSLP) and IL-2 production 23, 24. In contrast, S100A9 prevents the Th2-mediated asthmatic inflammation induced by *Alternaria alternata* extract in S100A9 knockout mice 25. Even though the knockout mouse model has a merit that target proteins are systemically depleted, it is difficult to determine alterations of the local inflammatory environment, including early eosinophilic and late neutrophil asthma stage, and to directly compare the mouse experiment with human study. Based on the results of the current study, we believe the role of S100A8 and S100A9 in the lung may be dependent on stimulators such as allergen, bacteria, and fungus.

TLR4 is an essential receptor binding to S100A8 and S100A9, and is associated with their proinflammatory response 1, 26. TLR4 is expressed in bronchial epithelial cells and mediates lipopolysaccharide-induced inflammatory responses 27, 28. The level of TLR4 is constitutively expressed in BEAS-2B cells and increases after treatment with S100A8 and S100A9 (Fig. 4). The alteration may augment cytokine production of S100A8 and S100A9 and TLR4-mediated inflammatory responses. Although TLR4 is a common receptor of S100A8 and S100A9, it has recently been reported that the function of S100A8 and S100A9 in acute lung injury is different 29. In the current study, S100A8 and S100A9 produce cytokines and suppress the normal and asthmatic neutrophil apoptosis (Figs 1, 5, and 6). This action is mediated by TLR4, PI3K, AKT, MAPKS, and NF-κB, and is similar to the inhibitory signal of neutrophil apoptosis due to S100A9 in monocytes (Figs. 2 and 3) 30. This study revealed no differences between functional mechanisms of S100A8 and S100A9. TLR4 functions as critical factors in neutrophilic asthma 31, 32. Our results imply that the signal of TLR4 mediated by S100 proteins is essential in severe or glucocorticoid-resistant neutrophilic asthma. S100A8, S100A9 and S100A12 secret MUC5AC in airway epithelial cells through TLR4 and RAGE 33. In addition, RAGE is another receptor of S100A8 and S100A9 34, 35. Although RAGE is not involved in acute lung injury induced by S100A8, its expression increases after exposure of S100A8 and S100A9 as alteration of TLR4 expression 36 (Supple. Fig. 2). Therefore, further studies on RAGE in bronchial epithelial cells and neutrophilic asthma patients will contribute to the relevant and additional evidence of the present study.

In conclusion, this study demonstrates that MCP-1, IL-6 and IL-8 are increased after exposure to S100A8 and S100A9 in BEAS-2B cells, through the PI3K/AKT/MAPK/NF-κB pathway. The increased cytokines delay constitutive neutrophil apoptosis. S100A8 and S100A9 trigger the interactive network between the BEAS-2B cells and neutrophils through cytokine secretion and apoptosis. We believe that these findings will pave the way for better understanding the pathogenesis of asthma.

## Supplementary Material

Supplementary figures.

## Abbreviations

DAMPs: damage-associated molecular patterns
ELISA: Enzyme-linked immunosorbent assay
FBS: fetal bovine serum
FITC: fluorescein isothiocyanate
PI: propidium iodide
RAGE: receptor for advanced glycation end products
TLR: Toll-like receptor
TSLP: thymic stromal lymphopoietin
