# Supplementary material for: Suppressive effects of S100A8 and S100A9 on neutrophil apoptosis by cytokine release of human bronchial epithelial cells in asthma
Source: Int J Med Sci. 2020 Feb 4;17(4):498–509. doi: 10.7150/ijms.37833 (PMC7053304; doi:10.7150/ijms.37833)
Supplement: Supplementary file 1 — Supplementary figures. [file ijmsv17p0498s1.pdf]

Supplementary figure legends

Supplementary Figure 1. Secretion of MCP-1, IL-6 and IL-8 is not altered by signal inhibitors in BEAS-2B cells

(A, B) BEAS-2B cells were treated for 48 h with or without 5 μM TLR4i, 5 μM LY294002 (LY), 10 μM AKTi, 10 μM PD98059 (PD), 10 μM SB202190 (SB), 10 μM SP600125 (SP) and 2 μM BAY-11-7085 (BAY). The supernatant (n=4) was collected and analyzed by ELISA. Data are expressed as the means ± SD, and are presented relative to the control set at 100%.

Supplementary Figure 2. Expression of RAGE in BEAS-2B cells after exposure to S100A8 and S100A9

BEAS-2B cells (n=3) were treated with 10 μg/mL S100A8 (A) and S100A9 (B) for the indicated time, and the expression of RAGE were analyzed by flow cytometry. Data are expressed as the means ± SD, and are presented relative to the control set at 1. *$p < 0.05$ and **$p < 0.01$ indicate a significant difference.

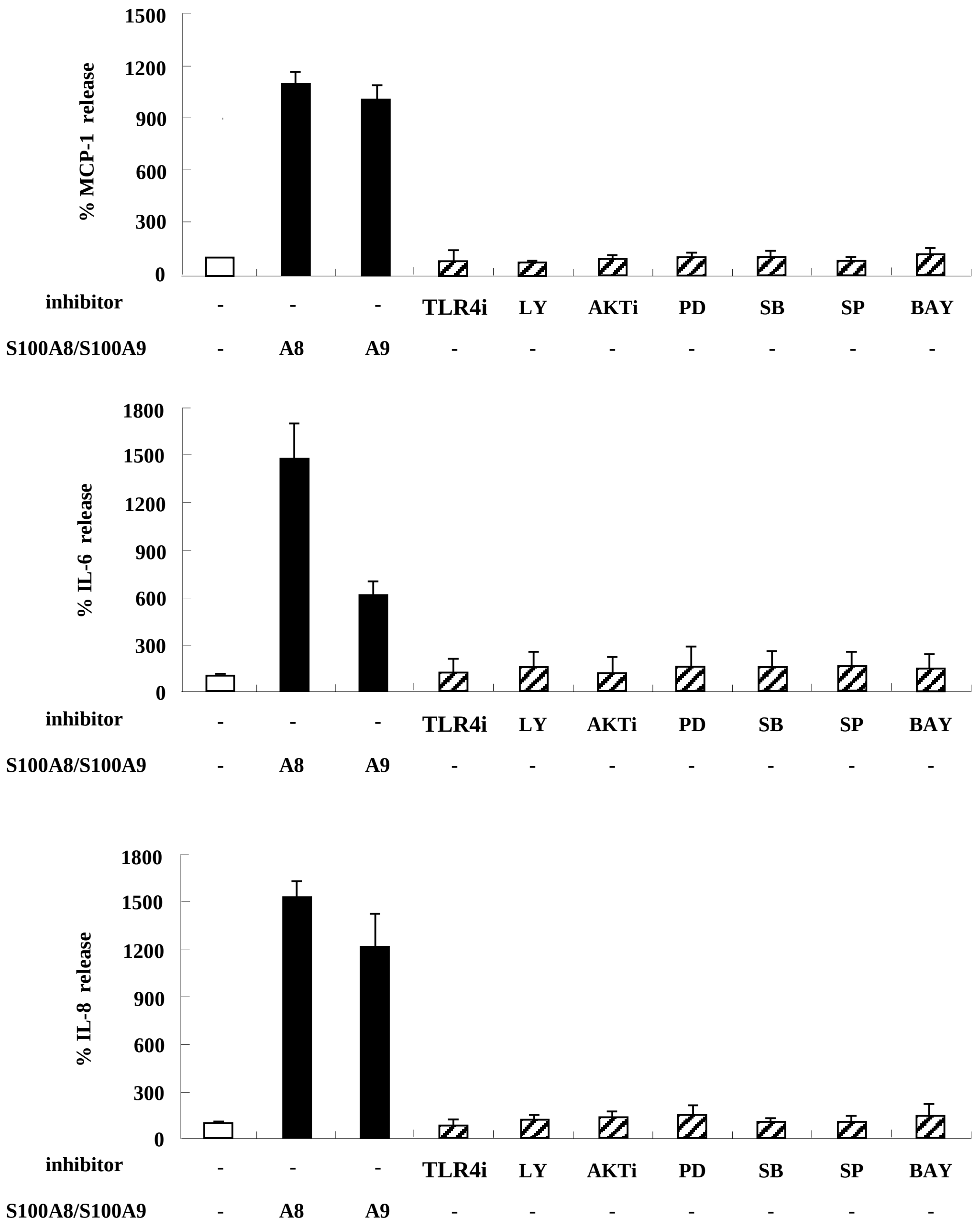

Supple Figure 1

A

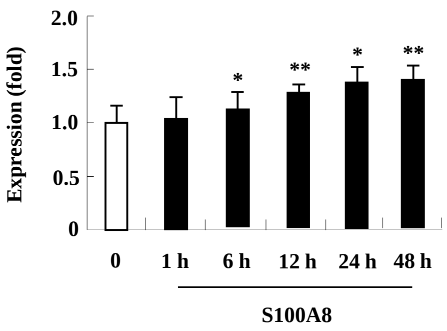

B

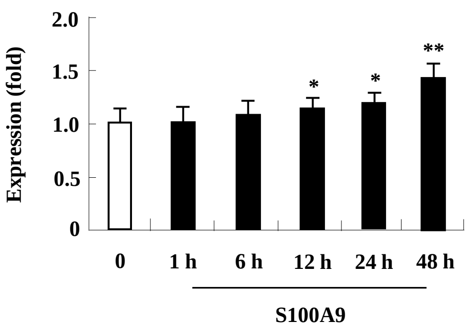

Supple Figure 2
